# Study of Charge Carrier Transport in GaN Sensors

**DOI:** 10.3390/ma9040293

**Published:** 2016-04-18

**Authors:** Eugenijus Gaubas, Tomas Ceponis, Edmundas Kuokstis, Dovile Meskauskaite, Jevgenij Pavlov, Ignas Reklaitis

**Affiliations:** Institute of Applied Research, Vilnius University, Sauletekio Ave. 9-III, Vilnius 10222, Lithuania; tomas.ceponis@ff.vu.lt (T.C.); edmundas.kuokstis@ff.vu.lt (E.K.); dovile.meskauskaite@tmi.vu.lt (D.M.); jevgenij.pavlov@tmi.vu.lt (J.P.); ignas.reklaitis@tmi.vu.lt (I.R.)

**Keywords:** materials for solid-state detectors, models and simulations, charge transport, polarization

## Abstract

Capacitor and Schottky diode sensors were fabricated on GaN material grown by hydride vapor phase epitaxy and metal-organic chemical vapor deposition techniques using plasma etching and metal deposition. The operational characteristics of these devices have been investigated by profiling current transients and by comparing the experimental regimes of the perpendicular and parallel injection of excess carrier domains. Profiling of the carrier injection location allows for the separation of the bipolar and the monopolar charge drift components. Carrier mobility values attributed to the hydride vapor phase epitaxy (HVPE) GaN material have been estimated as μ*_e_* = 1000 ± 200 cm^2^/Vs for electrons, and μ*_h_* = 400 ± 80 cm^2^/Vs for holes, respectively. Current transients under injection of the localized and bulk packets of excess carriers have been examined in order to determine the surface charge formation and polarization effects.

## 1. Introduction

Gallium nitride (GaN) is a promising material for application in particle and photo-detectors, due to its high radiation hardness and relevant operational characteristics [1,2,3,4]. Preliminary GaN-based sensors have been developed and promising results have been recently reported [5,6].

In this study, the structures of the capacitor and Schottky diode-type detectors have been fabricated on GaN material grown by metal-organic chemical vapor deposition (MOCVD) and hydride vapor phase epitaxy (HVPE) using plasma etching and metal deposition technology. In this article, the operational characteristics of detectors have been investigated by the transient current technique [5] using experimental regimes of perpendicular and parallel profiling. The experimental current transients have been correlated with simulated current transients by employing the dynamic model derived using Shockley-Ramo’s theorem [7,8]. The experimental regimes of injection of the localized and bulk excess carrier packets have been examined in order to establish the surface charge formation and polarization effects [9,10,11]. The screening effects have been observed and ascribed to surface charge, and to dynamics of extraction of the injected excess carriers. The obtained results are important for prediction of the functionality of GaN-based sensors. The role of the injected carrier recombination and drift, as well as the impact of polarization effects, has been revealed.

## 2. Samples and Experimental Regimes

GaN samples were grown on sapphire substrates by using HVPE and MOCVD techniques. The parameters of the examined samples are listed in Table 1.

**Figure 1:**
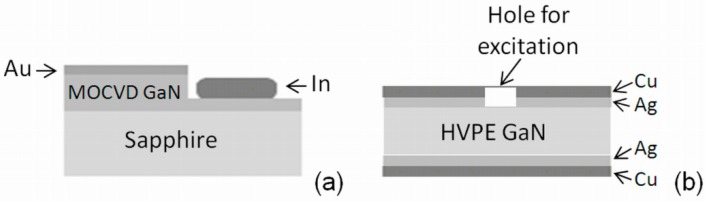
(**a**) Schematic sketches of the Schottky diode (SD); and (**b**) the capacitor C_d_ detectors.

The undoped u-GaN 24 µm thick epilayers were grown on sapphire substrates using a MOCVD reactor (Aixtron 3 × 2FT close coupled showerhead system [12]). A thin (~0.2 µm) buffer layer of n^+^-GaN was formed on the sapphire substrate during the growth of the MOCVD GaN epi-layer. This buffer layer serves as a layer of enhanced conductivity. Several semi-insulating (SI) GaN materials grown by HVPE technology and doped with compensating impurities were also employed for the fabrication of sensors. The GaN:Fe sample of a thickness of 475 µm, grown by the HVPE technique, was doped with Fe of concentration 2 × 10^17^ cm^−3^. Also, HVPE SI GaN samples of a thickness of 400 µm contained growth defects and were doped with other compensating metallic impurities of low (HVPE-LD, ~10^16^ cm^−3^) and high (HVPE-HD, ~10^19^ cm^−3^) concentration. The capacitor (C_d_) and Schottky diode (SD) type detectors have been fabricated using MOCVD GaN, whereas the capacitor sensors were made of HVPE SI GaN free-standing wafer fragments. The capacitor-type mesa structure detectors were fabricated using plasma etching. The electric contacts were formed by using pressed Cu plate electrodes and Ag paste contact deposition on a cleaned sample surface. The Schottky barrier-type mesa detectors were formed by deposition of the sintered In cathode at the bottom terrace, which was etched on a GaN epi-layer, and the anode was fabricated by Au sputtering on top of the mesa structure. The schematic views of the fabricated structures are illustrated in Figure 1. The top opening of ~1.5 mm width (significantly prevailing wafer thickness to suppress the role of lateral diffusion of the light-injected carriers) was made for excitation of excess carriers.

**Figure 2:**
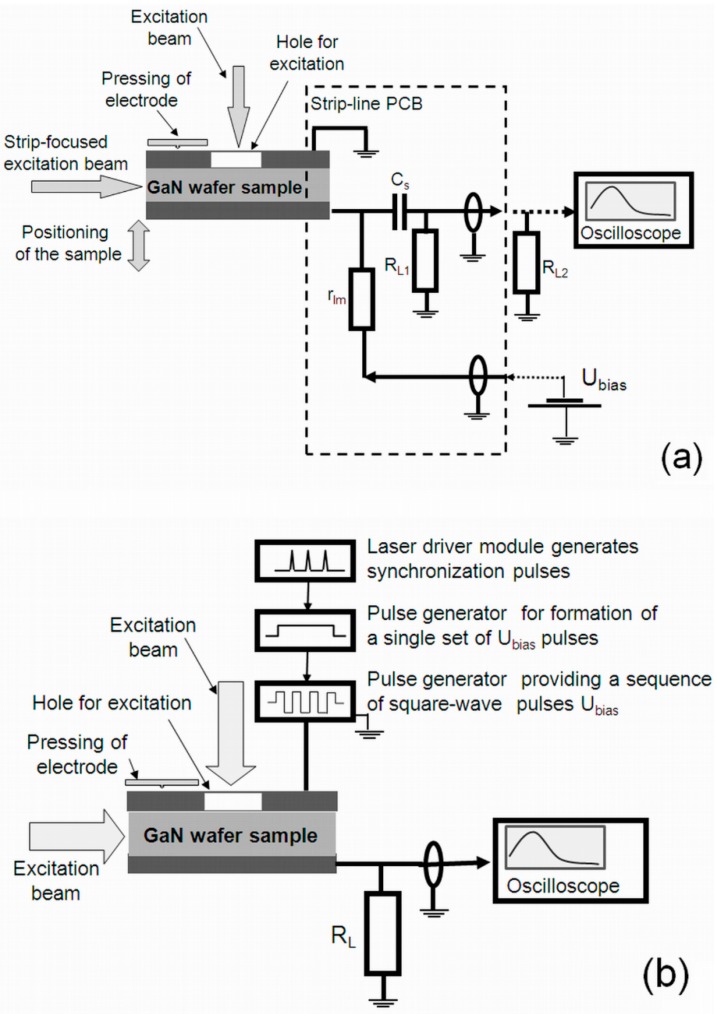
(**a**) Arrangement of measurement circuitries for the profiling of the carrier packet injection location; and (**b**) for examination of the polarization relaxation.

The experimental setups exploited for recording of the pulsed current and the polarization relaxation transients are shown in Figure 2a,b, respectively. More details on experimental arrangements can be found elsewhere [13,14,15,16]. The sensor was mounted on a strip-line (to exclude frequency limitations for signal transfer within external circuit), implemented as a printed circuit board (PCB). The load resistance *R*_L,1||2_ = 25 Ω (Figure 2a) was employed for recording of the rather short pulses. The excess carrier packets (domains) were injected by using a beam of 354 or 532 nm light laser pulses of τ_L_ = 400 ps in duration and with a 100 Hz repetition rate. A laser beam was focused by a cylindrical lens to a Δ = 10 µm strip, which covered all of the sample length to minimize the impact of the side diffusion of light-injected carriers. The side diffusion of carriers complicates dynamics of electric field distribution, when a one-dimensional approach becomes invalid. The sample depth over the inter-electrode gap was scanned by moving the sample relative to this focused light strip. The energy of <1 µJ per laser pulse was kept, to implement the regime of small charge drift as well as to avoid screening effects attributed to the injected excess carriers, and the excitation intensity was lowered by neutral filters.

GaN material is a binary compound containing a crystalline structure with a lack of inversion center symmetry [9,10,11,17,18,19,20]. Thus, a GaN crystal has a built-in polarization ascribed to the piezoelectric and the spontaneous polarization components. This polarization is directed along the crystal growth c-axis (0001), perpendicular to the epi-layer surface. For the bulk GaN material in equilibrium, this polarization field is screened by free carriers excited through band tilting and generated from the impurity and defect states. This built-in polarization can be a reason for the appearance of a steady-state surface charge if the mobile carriers are extracted from the bulk by the applied external field. This built-in charge is able to screen the charge on the device electrodes induced by an external source. Free carriers trapped on the deep levels can also create a localized space charge within a region close to the electrode. Surface reconstruction during crystal relaxation may also be a reason [18] for suppressed crystal polarization effects in bulk material samples. Finally, the extraction of a sub-system (of electrons or holes) of the light-induced excess carrier pairs may be a reason for the formation of charged layers. These layers are depleted from one of the excess carrier sub-system, as injected electrons and holes move to opposite electrodes. There, the electrically neutral region appears between the depleted layers, due to the sufficient density of excess carrier pairs to screen the external field (surface charge on electrodes). Thus, the acting electric field appears as a superposition of the fields. These fields are created by the surface charges on electrodes induced by an external battery, by the mobile charges of the light pulse-injected carrier pairs, by the localized charge of carriers captured at deep traps and by the surface charge due to the built-in crystal polarization. The polarization effect may then occur due to the mentioned steady-state and dynamic components.

Different regimes of profiling of the sensor signals can be implemented by varying injection position and applied voltage to clarify the role of these components and to extract the parameters of carrier transport. Forming the local domains of drifting charges can be performed using carrier injection via pulsed excitation of the carriers through a cross-sectional boundary of a sensor structure when the excitation beam is oriented perpendicularly to the acting electric field. The profiling of sensor current pulses can also be implemented by varying voltage that is able to extract a different amount of the injected carriers, which are excited within the entire inter-electrode gap (where the excitation beam impinges in parallel to the acting electric field).

In MOCVD GaN mesa structure devices and HVPE GaN wafer fragments employed for fabrication of the capacitor-type and Schottky barrier sensors, the external field was always oriented along the c-axis. Therefore, for the range of rather low applied voltages, the polarization can suppress the external field, and the drift current signals disappear, similarly to the formation of virtual gate and drain current collapse effects in field effect devices [20]. To reveal the dynamic and the steady-state components of the polarization, measurements of the relaxation of polarization were additionally performed. A recovery of the external field governed current transients can be observed by either switching off the voltage or by the sudden changing of the voltage polarity. To highlight the changes of the charge induced by the external voltage source, the transformations of the capacitor charging transient after each laser pulse (which injects carrier pairs and thus modifies the role/state of the surface polarization) have been examined. This has been implemented by using long pulses of applied voltage synchronized with the laser pulses which inject excess carrier pairs. Several pulse generators, sketched in Figure 2b, were employed to arrange a single voltage pulse or a set of the square-wave bipolar voltage pulses. A set of laser light pulses generates the excess carriers in the bulk of GaN device during the voltage pulse. There, excitation was performed by impinging a laser beam either through a hole made within electrode or by illuminating a cross-section of a device, as sketched in Figure 2b. The set of the short charging current pulses of the amplitude decreasing in time is then recorded (as vertical lines) within a timescale of a voltage pulse and a set of laser pulses running with 100 Hz repetition rate. These oscillograms directly represent a transient of suppression and recovery of the drift current values that were reduced due to accumulated polarization charge.

## 3. Models for Analysis of Current Transients

The simulation models for analysis of current transients, determined by drift-diffusion processes, have been developed based on Shockley-Ramo’s theorem [7,8]. Current temporal variations are considered to be governed by the carrier generation/recombination and drift/diffusion processes. The current depends on the injection position of the moving carrier packet within the inter-electrode gap. The detail description of these models, applied to the capacitor type and diode detectors, is published in Refs. [13,14,15,16].

The transients are recorded within an external circuit, therefore, the current is described as the surface charge density σ(*t*) variations in time integrated over an area *S* of electrode. Under a switched-on external DC voltage *U*, a surface charge on electrodes is initially induced by an external battery. An additional surface charge on electrodes might be induced by the spontaneous polarization of GaN material [9,10,11], filling the inter-electrode gap, as contacts are deposited within plane perpendicular to the c-axis of the MOCVD/HVPE grown crystal. The equilibrium/steady-state polarization charge can nevertheless be ignored in bulk GaN material, as the surface relaxation and reconstruction [18,19,20] of the GaN sample and defects in the material lead to compensation of this polarization surface charge. The current transient is then formed by the injection/recombination and drift/diffusion of a light pulse-injected domain of excess carrier pairs (*e-h*) with surface charge density (*q_e_*, *q_h_*). It is assumed that the injected surface charge domains contain the same area as contacts, and that the electric fields created by these mobile domains are oriented in parallel to electric field induced by an external source. These surface charges (*q_e_*, *q_h_*) (due to their motion or changes in density) induce surface charges on electrodes (according to Shockley-Ramo’s theorem). Under the assumption of parallel oriented electric fields created by surface charges on electrodes and of injected domains, the moving surface charge domains create the varied electric fields *E*_q_(*t,***ψ**) = *q***ψ***(t)/*εε_0_ dependent on the instantaneous position *X_q_* of the mobile domains within inter-electrode gap *d*, expressed through dimensionless parameter **ψ***(t) = X_q_(t)/d*. Thus, a superposition of the electric fields, created by the mobile surface charge domains and those of external battery, determines variation of surface charge on electrodes. The invariable voltage drop on sensor (as an integral of acting electric fields over inter-electrode gap *d*) is retained by changes in battery-induced charge on electrodes. These charge variations (as a battery’s feedback to field variations within a sensor due to the motion of the injected carriers) are namely registered as a current transient within an external circuit, *i.e.*, on load resistor.

The generalized expression for the current dependence on time (*t*) ascribed to the bipolar (*e-h*) drift processes, as well as to the instantaneous positions of sub-domains (**ψ***_e,h_*(*t*)), governed by the carrier drift and capture processes, reads as

(1) $$i(t)=\frac{d\sigma }{dt}S=[-\frac{\partial q_{e}(t)}{\partial t}(1-\psi_{e}(t))-q_{h}(t)\frac{d\psi_{h}(t)}{dt}-\frac{\partial q_{h}(t)}{\partial t}\psi_{h}(t)-q_{h}(t)\frac{\partial \psi_{h}(t)}{\partial t}]S$$

This current contains the drift stages for the both polarity (bipolar) charges, which may be prolonged by the monopolar drift of holes (or electrons), if transit times (for e and h sub-domains) differ. In the case of hole monopolar drift, the current components are expressed as

(2) i(t)={i1=qSτtr,e[ψexp0(−tτtr,e)+(1−ψ0)], for 0≤t≤τtr,e=τbC;  i2=qhSτMq,hexp(tτMq,h)[v0,ΣbipdτMq,h+τMq,hτTOF,h−1], for 0≤t≤τtr,h≡τtr,h,mon 

here, τ*_tr_*, τ*_Mq,e,h_*, and τ*_TOF,e,h_* denote the characteristic times of transit, of dielectric relaxation and of free flight, respectively. Other symbols represent the following: *v*_0_ is the initial velocity of the monopolar charge drift, *d* is the inter-electrode gap width, **ψ**_0_ and **ψ***_e,h_* are the dimensionless locations of the initial injection and of the instantaneous positions of drifting packets, respectively. The analytical description of the current transients, at low applied voltages *U* and bulk injection of an electron-hole packet, is implemented by considering the instantaneous position *X_e,h_(t)* = **ψ***_e,h_(t)d* variations. There appears the characteristic depth *X_e,h_*_0_ of depletion which, in the case of the electron extraction, is represented as

(3) X(n0,U)e0=d2(1−1−εε0Uen0(d2)2)≈d2(εε0(U/2)en0(d2)2)

The depletion layers close to the surface occur due to screening/balance of the battery-supplied surface charge by the charge of excess carriers left after the initial extraction of their counter-partners within the light-injected domain. The current variation in time (*t*) during the initial stage of a pulse (*i*_1_(*t*)), when injected carriers drift and the depletion layer *X_e,h_*_0_ forms, is obtained as

(4) $$i_{1}(t)=S\frac{d\sigma }{dt}=\frac{S}{X_{e}(t)}\left(\frac{\varepsilon \varepsilon_{0}U}{X_{e}(t)}-en_{0}X_{e}(t)\right)\frac{dX_{e}(t)}{dt}$$

Here, ε_0_ and ε represent permittivity of vacuum and material, respectively, and *n*_0_ is the initial concentration of the injected carrier pairs. A width of depletion layers can be increased and a reduction of the current within the initial stage of transients can occur for a set of injection light pulses applied if an accumulation of the non-extracted carriers takes place. This may be a reason for sensor polarization. The accumulated carriers then prevent further induction of charge from the battery. This leads to a collapse of current (*i*_1_) in transients generated by the set of injection light pulses. The current (*i*_1_) value can only be restored if these mobile accumulated carriers are extracted by reverse polarity of the battery or shunted by a resistor installed in parallel to a sensor. These (near-surface) accumulated carriers determine the dynamic polarization effect. Certainly, the accumulated carriers can also be trapped to deep levels residing in the near-surface region (for instance, due to dangling bonds, impurities, *etc.*). This forms a space charge region which also causes polarization of the sensor. The latter polarization can be reduced after trapped carriers are thermally emitted to the continuum states and extracted by the appropriate polarity of the external voltage (or under relaxation to equilibrium after external voltage source is switched off). Both of the dynamic components of sensor polarization determine the current reduction (within the transients generated by a set of injection pulses) and duration of sensor recovery (polarization relaxation).

Using equation for the instantaneous drift velocities (d*X_e_/*d*t*) of carriers under acting electric fields (expressed through the dimensionless instantaneous locations (**ψ***(t*)) of drifting charges), the initial stage (for the time interval 0 ≤ *t* ≤ τ*_tr_*) of the drift current pulse can be described as

(5) $$i_{1}(t)=\frac{S\varepsilon \varepsilon_{0}}{\psi (t)d}U\frac{1}{2\tau_{Mn0}}\left[\frac{1}{\psi (t)}-\frac{\tau_{TOF}}{\tau_{Mn0}}\psi (t)\right]\times \left[1+\psi (t)-\frac{\tau_{Mn0}}{\tau_{TOF}}\frac{1}{\psi (t)}\right]$$

An additional current component, ascribed to the carrier capture and recombination (with inherent lifetimes τ*_C_* and τ*_R_*, respectively), is introduced as

(6) $$i{\left(t\right)}_{+\tau_{R}}=\{\begin{array}{c} i_{1}(t){|}_{0\leq t\leq \tau_{tr}}-eS\frac{\partial (n_{0}e^{-\frac{t}{\tau_{R}}})}{\partial t}X_{e}(t)\\ i_{2}(t-\tau_{tr}){|}_{t_{2}>\tau_{tr}}+eSd_{eff}n_{0}(t=0)e^{-\frac{t_{2}}{\tau_{R}}}\sum_{k=0}^{\infty }\left(\frac{8}{\pi^{2}}{\left(-1\right)}^{k}\frac{\exp\left(\frac{4D_{a}\pi^{2}}{d_{eff}^{2}}{\left(2k+1\right)}^{2}t_{2}\right)}{{\left(2k+1\right)}^{2}}[\frac{1}{\tau_{D,k}}-\frac{1}{\tau_{R}}]\right) \end{array}$$

Here, *d_eff_ = d − X_e_*_0_ *− X_h_*_0_, τ*_D,k_* = *d_eff_*^2^/(4π^2^*D_a_*(2*k* + 1)), and *D_a_* is the coefficient of carrier ambipolar diffusion. More detail derivations of Equations (1)–(6) can be found in [13,14,15,16].

Solving the current equation for the external circuit with a characteristic relaxation time *R_L,_*_1*||*2_*C_d,SD_*, the actual current transient shape (slightly modified the idealized transient, described by Equations (1)–(6), due to delays within external circuit) can be simulated and employed for the fitting of the experimental transients.

## 4. Experimental Results and Discussion

### 4.1. Recombination Characteristics

**Figure 3:**
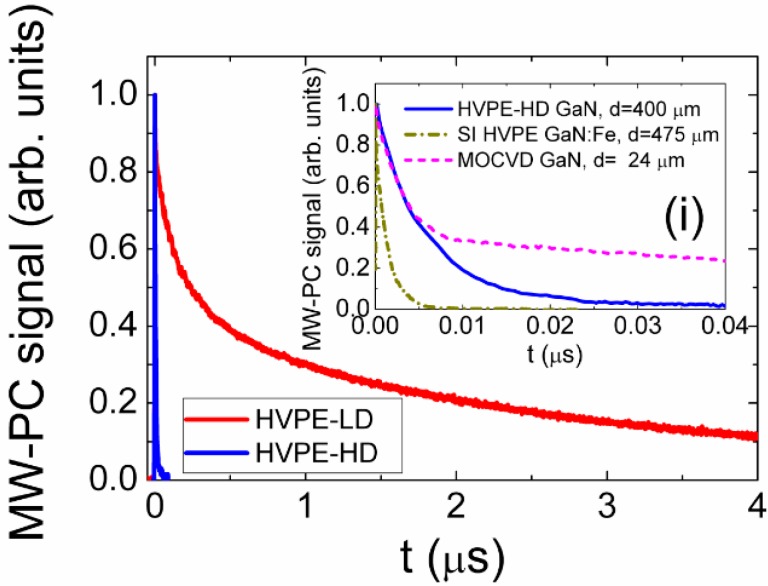
Microwave probed photo-conductivity (MW-PC) transients in HVPE and MOCVD GaN material.

The comprehensive study of the recombination and of the device characteristics has been performed by combining the analysis of the microwave probed photo-conductivity (MW-PC) [21,22] and of the detector current transients. MW-PC transients, recorded without an external electric field, showed a wide variety in recombination and trapping lifetimes (Figure 3).

The long-tail non-exponential behavior of the MW-PC transients (more detail discussion is presented elsewhere [23]) is caused by excess carrier trapping and de-trapping due to defects that are commonly introduced during MOCVD GaN crystal growth. The MW-PC transients obtained from MOCVD samples can be described by stretched-exponential relaxation (SER) [24,25], indicating the significant disorder induced by defects in the material. Spectroscopy data for defects in GaN employed for fabrication of the device structures are presented in our article [26].

Excess carrier relaxation transients (Figure 3) in semi-insulating HVPE GaN samples showed considerably different time scales. It has been found that carrier recombination lifetime decreases with the enhancement of the concentration of the compensating metallic dopants, which also act as recombination centers.

### 4.2. Profiling of Current Transients in HVPE GaN Sensors

**Figure 4:**
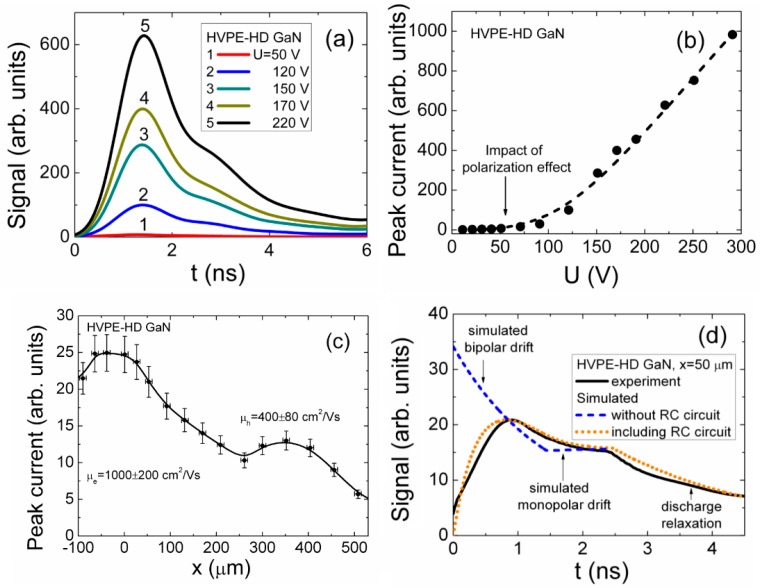
(**a**) Profiling of current transients by varied applied voltage; and (**b**) peak amplitudes as a function of applied voltage; (**c**) Profiling of current transients by varying location of the initial injection of electron-hole (*e-h*) domain in HVPE GaN; and (**d**) the solid curve represents the transient as recorded within cross-sectional scan for carrier injection location at *x* = *X*_0_ = 50 µm; the dashed curve represents the simulated current transient without including the impact of the external circuit; the dotted curve simulated with including delays within the external circuit.

Two experimental regimes have been implemented for profiling of current transients: (i) by varying the applied voltage when an excitation beam is impinging in parallel to the external battery created electric field; and (ii) by varying the location of a focused injection beam within the inter-electrode gap when a laser beam is perpendicular to the electric field. The results of profiling of the injected charge current transients at room temperature for an HVPE-HD sample with a high concentration of dopants are illustrated in Figure 4. The shape of the current transients is entirely related to the predominant process of recombination and drift-diffusion of the excess carriers, described in Section 3.

The primary current peak (*i_peak_(t)* = *q/*τ*_tr,bip_*) in voltage profiled transients (Figure 4a) is attributed to the bipolar drift of carriers, while a component of the decreasing current can be ascribed to the excess carrier trapping/recombination. The peak amplitudes of the current pulses dependent on detector biasing voltage show the impact of the polarization effect (Figure 4b). The formation of the near-surface polarization charge by the injected excess carriers prevents the charging of the device by the external battery of small voltage. These injected carriers may create a depletion layer either due mobile carriers (they cannot be extracted by a fixed polarity of external field), or they can be trapped by defect levels forming a localized space charge. This space charge may screen the electric field created by an external battery. Thus, the polarization charge suppresses the control of the injected carrier current by a small applied voltage (<50 V, Figure 4b). This voltage profiling of current transients, ascribed to the injected bulk domains of carriers covering the entire inter-electrode gap, is suitable for the evaluation of the role of the polarization effects. The current increase with voltage is clearly observed only for the voltages exceeding a threshold value of *U_th_*_r_ ≥ 100 V (Figure 4b). The current then increases almost linearly (Figure 4b), with external voltage (>100 V) due to shortened transit time at elevated voltages for bipolar drift of injected carriers. However, this profiling regime does not allow to definitely evaluate the drift distance and transit time, as carrier trapping/recombination controls a rear stage of the current transient when the transit time is close to the carrier lifetime.

The carrier mobility values have been estimated by an analysis of the current dependence on locations of the electron-hole (*e-h*) domain injection (Figure 4c,d), where drift distances of electrons and holes can be discriminated. For extraction of carrier mobility, the drift distance for the injected carrier sub-domain (which first reaches a contact) is evaluated from the injection position (within a profile of the peak current as a function of injection location) by including the polarity of the applied voltage. The polarity of the applied voltage determines a sign (charge polarity) of the collected carriers, those arriving first to the electrode. The transit time for each injection position is evaluated from the minimal current value within the respective (relative to each fixed position of injection) current transient (shown by dashed curve in Figure 4d). The transit times coincide for both carrier sub-domains within a stage of bipolar drift in Figure 4d, however, the drift distances are different due to different carrier mobilities. The drift time for bipolar motion stage is determined by transit of the sub-domain, which first reaches the contact, as sub-domains synchronously move in opposite directions (Figure 4d). The surface charge induced on electrodes by the latter sub-domain modifies electric field acting on another sub-domain. Therefore, the current minimum is reached at the end of the bipolar drift for a definite current transient (Figure 4d). On the other hand, the minimum current at the end of the bipolar drift phase can be ascribed to the longest drift path and to a consequent transit time for a late sub-domain within the pure bipolar drift process (Figure 4d). The flat or the ascending vertex of the second peak within current pulse indicates the monopolar drift. The transit time ascribed to the monopolar drift is then evaluated as the time interval between an instant where minimal current is reached and the end of the current pulse. The end of a drift pulse is estimated by the current bend within rear phase of a transient. The experimental transients are more complicated due to the delays, caused by external circuit elements, and the carrier capture/recombination processes. The durations of the current rise to peak and of relaxation may be distorted by the delays within the external circuit and carrier capture. Therefore, for the more precise extraction of drift parameters under mentioned complications, simulation of the current transients recorded for each injection location (Figure 4d) is correlated with the simulation of the entire profile (Figure 4c) for the peak current dependence on injection position.

The current minimum can also be observed within a profile of peak current dependent on injection position (Figure 4c). This current minimum is obtained for the peculiar injection position when both sub-domains run the longest distances and reach electrodes synchronously. The square-wave shape transient would be an indication of such a pure bipolar drift regime, observable for a small density of drifting carriers when carrier capture can be excluded. The minimal current value is obtained (Figure 4c), as current is a reciprocal function of the transit time. Indeed, a minimal current is observed (Figure 4c) within a peak current profile, measured relative to injection position.

By combining the analysis of transients and profiles (Figure 4c,d), the drift distances and transit times are evaluated and employed for extraction of carrier mobilities. To reproduce the transit time components, the recorded transients are fitted by simulated ones (as illustrated in Figure 4d for a transient recorded for a fixed injection location) using the dynamic model (sketched by Equations (1)–(6)). Simulations are performed for every definite injection location using the parameters of the fixed external circuit (*U*, *C_d,SD_*, *S*, *R_L_*) as well as of the injection (*n*_0_, *X*_0_, Δ, *D_a_*, τ*_C_*, τ*_L_*). The initial stage of the current pulse is always determined by the bipolar charge drift. However, the rear component of a transient is always ascribed to the monopolar charge drift, as follows from the dynamic models for small charge drift. The shape of the latter rear component depends on the applied voltage and on the density of the injected excess carrier pairs, resulting either in the rising current shape or the forming of a rather flat vertex. The polarity of charge involved into the monopolar drift is unambiguously defined by the polarity of the external voltage. The polarity and value of the applied voltage, the transit times extracted from transients recorded for each injection location (by fitting experimental and simulated data) and the parameters of the external circuit (as RC_d_) were taken into account for the evaluations of carrier mobility. Actually, the injection location and transit times for bipolar and monopolar drift were adjusted with relevant mobility of sub-domains in fitting of the experimental transients for evaluation of carrier mobilities. It is evident that the simulation procedure should be performed within a multi-dimensional space of the parameters of the external circuit, of the injection and of the material (µ*_e_*, µ*_h_*). Therefore, the most possible quantity of the independent relations (equations) is desirable. The peak current profile, illustrated in Figure 4c, provides the additional relation to a set of transients, of the type illustrated in Figure 4d. For the precise extraction of the material parameters (e.g., ascribed to carrier drift: µ*_e_*, µ*_h_*), a lot of iteration procedures should be performed by minimizing a functional in the non-linear least square method, serving as a measure for the best fitting between the experimental and simulated data.

Values of carrier mobility have been obtained to be μ*_e_* = 1000 ± 200 cm^2^/Vs for electrons and μ*_h_* = 400 ± 80 cm^2^/Vs for holes, respectively. The statistical errors for extraction of mobility values are in the range of 20% relative to mean values. These errors are mainly arisen from uncertainties due to positioning of injection beam and simulation errors in evaluation of transit times. The obtained electron mobility value is in agreement with published data [27,28,29,30], while the estimated hole mobility value is twice less than those reported in [31], although it agrees with value reported in [32].

### 4.3. Current Transients in GaN Materials with Short Carrier Lifetime

The discussed observations also imply the significant role of carrier capture in materials of short carrier lifetimes, in the range of a few ns, as revealed for HVPE GaN:Fe and MOCVD GaN materials. There, the generation/carrier capture current components prevail.

**Figure 5:**
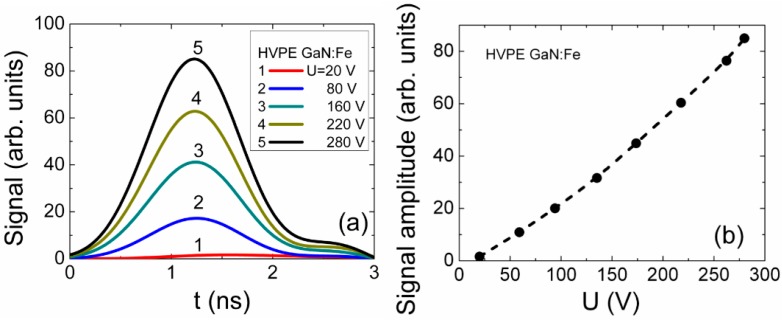
(**a**) Profiling of current transients; (**b**) peak amplitudes by applied voltage in HVPE GaN:Fe.

For the semi-insulating GaN material, *i.e.*, HVPE GaN doped with Fe, current pulse duration (Figure 5a) appeared to be shorter than that for the unintentionally doped HVPE GaN sample, as can be deduced by comparing transients in Figure 4a and Figure 5a. Additionally, for the GaN:Fe sample where short recombination lifetimes (of τ*_R_* ~ 2 ns) were deduced from MW-PC transients, the peak current increases linearly (Figure 5b) with applied voltage over the entire range of the applied voltages, *i.e.*, the polarization effect is nearly negligible. In this GaN:Fe material, the current pulses, profiled by varying the injection location, appeared to be significantly shorter than those transients recorded (Figure 4d) for the unintentionally doped HVPE GaN material with a longer carrier lifetime. The current increases with enhancement of voltage (Figure 5a) due to the shortened transit time ascribed to the bipolar drift. However, due to rapid capture of carriers, this bipolar drift phase is not completed, carriers disappear (through recombination) before one of the drifting sub-domains reaches a contact. This type of drift current is similar to that described by Hecht’s model [33]. This can alternatively be understood as an increase of the excess carrier portion involved in drift. The registered sensor signal represents a sum of carrier drift and generation/capture current components (included within Equation (6)). Thereby, the current pulse duration is mainly determined by carrier lifetime. The transit time is indefinite in this case, even for the bipolar drift phase, as neither drift distance nor drift duration can reliably be extracted from the recorded transients of 1–2 ns duration. In this case, the current pulse duration is close to the carrier lifetime and the carrier drift parameters are hardly measurable in the materials with short carrier lifetime. The peak current increase with voltage is then obtained to be close to a linear function (Figure 5b), as a rather small concentration of excess carriers is involved in drift. The carrier generation/capture current component then prevails in the formation of the transient duration, while the current component attributed to the bipolar drift increases with voltage. The observed pulse durations are in good agreement with carrier recombination lifetimes measured by the MW-PC technique. The peak current (Figure 5b) increases linearly with voltage due to predominant carrier drift processes. There, the peak current is an inverse function of a drift time, which decreases almost linearly with the enhancement of voltage.

### 4.4. Polarization Effects

**Figure 6:**
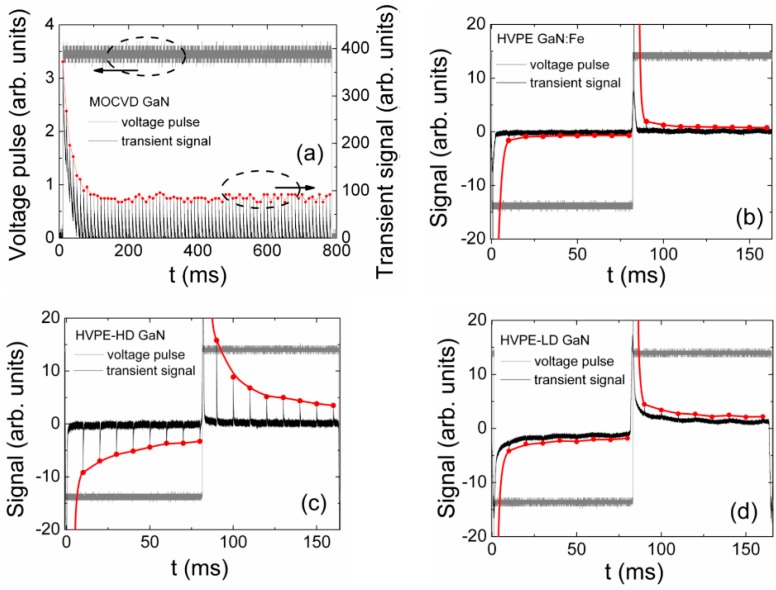
(**a**) Relaxation of peak current values under a set of injection light pulses in MOCVD GaN Schottky structure; (**b**) in HVPE GaN capacitor type sensors structures (sample of compensated GaN:Fe; (**c**) and GaN samples HVPE-HD; (**d**) HVPE-LD due to polarization effect. Dashed ellipses indicate the groups of data points (curves) those are related with coordinate axis denoted by arrows.

To clarify the dynamics of polarization effect in MOCVD and HVPE GaN samples, the relaxation processes have been examined. This type of relaxation characteristic has been measured by controlling the changes of the amplitudes of the charging currents under action of a set of light pulses keeping either the invariable switched-on DC voltage or unipolar voltage pulse (Figure 6a) for the MOCVD GaN and by varying polarity of applied voltage for the HVPE GaN (Figure 6b–d).

The formation of the surface charge, and thus the polarization of the MOCVD GaN structures, seems to be caused by carrier capture into deep levels and the formation of the localized space charge within near-surface regions, as discussed in Section 3. The characteristic relaxation time on the order of a hundred milliseconds (Figure 6a) represents the duration of a balance between carrier capture to deep traps and thermal emission of trapped carriers in the formation of a depletion layer of localized space charge capable of screening the external field. The relaxation transients of polarization appeared to be dependent on a series resistance for the MOCVD GaN capacitor-type and Schottky diode structures. The series resistance in MOCVD devices appears due to the inappropriate quality of contacts deposited on the mesa structure. The series resistance leads to an increased offset within the initial component of the relaxation curve in Figure 6a.

For the HVPE GaN capacitor-type sensors, the relaxation curve shape and duration depends on the doping level of the material (Figure 6b–d). The initial offset for the relaxation curves (Figure 6b,c) is rather small in highly doped GaN:Fe and HD GaN material sensors due to the short carrier capture time. The duration of polarization charge creation during the formation of depletion layers correlates well with carrier lifetimes when comparing transients in Figure 6b (for GaN:Fe) and 6c (for HD GaN), where carrier lifetimes of τ*_R_* ≤ 2 ns in GaN:Fe and τ*_R_* ~ 20 ns in HVPE-HD GaN, respectively, were measured by MW-PC. Thus, the manifestation of the material polarization depends on the bulk concentration of dopants and of excess carriers that are able to create polarization charge that screens the external field. Additionally, two relaxation components, fast and slow, can be clearly discriminated for the polarization process within the curves of Figure 6c. The characteristic times on the order of tens and hundreds of milliseconds ascribed to the slow component correlate with the time scale of thermal generation lifetimes attributed to deep levels. Consequently, the variation of the sensor signals (Figure 4a–d) determined by the injection of the excess carrier pairs and applied voltage is also sensitive to the time scale of formation/suppression of polarization.

The recorded peak amplitudes of the charging current pulses also depend on the doping of the HVPE GaN material (Figure 6c,d). This observation can be explained by different density of impurities and crystalline defects that modify the material ability either to accumulate the trapped carriers, by forming surface charge, or to modify the built-in polarization. The density of defects also modifies the rate of the excess carrier decay. As a consequence, the polarization effect exhibits the steady-state (slow) and dynamic (fast) components for the HVPE GaN sensors. The charge collection efficiency realized at low voltages for injection of the bulk excess carrier domain, covering the entire inter-electrode gap, can be considerably limited due to the screening of the external electric field by a surface charge (polarization field) in such structures. The excess carriers residing nearby the electrodes can only be collected during the formation of the depletion layers at low voltages. The excess carrier pairs deep in the bulk aggregate an electrically neutral region which separates the depleted layers at electrodes.

## 5. Summary

The GaN based capacitor and Schottky diode-type detectors have been fabricated. Two regimes have been implemented for the profiling of current transients in these devices: (i) by varying the applied voltage when an excitation beam is parallel to the electric field; and (ii) by varying the location of a focused injection beam within the inter-electrode gap when a laser beam is perpendicular to the electric field. The carrier capture and drift components, as well as the polarization effect, have been revealed in different GaN samples. It has been shown that the role of the polarization effect decreases with the enhancement of the applied voltage. The drift component can be well discriminated by varying a location of a focused injection beam within the inter-electrode gap when the local e-h domains are injected. The profiling of the carrier injection location allows for the separating of the bipolar and monopolar drift components. The drift distance for drift of each sub-domain and their transit times can then be discriminated and evaluated. Using the parameters of the drift paths and of the transit times, the carrier mobility has been extracted. Carrier mobility values attributed to the HVPE GaN material have been estimated to be μ*_e_* = 1000 ± 200 cm^2^/Vs and μ*_h_* = 400 ± 80 cm^2^/Vs for electrons and holes, respectively. The relaxation of the peak current values due to the polarization effect has been examined using a set of the excitation pulses. For the HVPE GaN capacitor-type sensors, the relaxation curve shape and its duration depends on the doping of material. The polarization effect exhibits the components of the steady-state (slow) and of the dynamic (fast) polarization. The charge collection efficiency at low voltages can be considerably limited due to the screening of the external electric field caused by the polarization effects in such structures under injection of the bulk charge within the inter-electrode gap.

## Figures and Tables

**Table 1:** Parameters of the samples fabricated as the capacitor (C_d_) and Schottky diode (SD) devices. Hydride vapor phase epitaxy: (HVPE); metal-organic chemical vapor deposition: (MOCVD).

GaN Sample	Thickness (μm)	Technology	Device Structure
u-GaN	24	MOCVD	C_d_ and SD
SI GaN:Fe	475	HVPE	C_d_
HVPE-LD	400	HVPE	C_d_
HVPE-HD	400	HVPE	C_d_
